# Embodied airflow sensing for improved in-gust flight of flapping wing MAVs

**DOI:** 10.3389/frobt.2022.1060933

**Published:** 2022-12-07

**Authors:** Chenyao Wang, Sunyi Wang, Guido De Croon, Salua Hamaza

**Affiliations:** BioMorphic Intelligence Lab & Micro Air Vehicle Lab, Faculty of Aerospace Engineering, TU Delft, Delft, Netherlands

**Keywords:** flapping wing MAV, bio-inspired sensing, adaptive control, in-gust flight, onboard airflow sensing

## Abstract

Flapping wing micro aerial vehicles (FWMAVs) are known for their flight agility and maneuverability. These bio-inspired and lightweight flying robots still present limitations in their ability to fly in direct wind and gusts, as their stability is severely compromised in contrast with their biological counterparts. To this end, this work aims at making in-gust flight of flapping wing drones possible using an embodied airflow sensing approach combined with an adaptive control framework at the velocity and position control loops. At first, an extensive experimental campaign is conducted on a real FWMAV to generate a reliable and accurate model of the in-gust flight dynamics, which informs the design of the adaptive position and velocity controllers. With an extended experimental validation, this embodied airflow-sensing approach integrated with the adaptive controller reduces the root-mean-square errors along the wind direction by 25.15% when the drone is subject to frontal wind gusts of alternating speeds up to 2.4 m/*s*, compared to the case with a standard cascaded PID controller. The proposed sensing and control framework improve flight performance reliably and serve as the basis of future progress in the field of in-gust flight of lightweight FWMAVs.

## 1 Introduction

### 1.1 Airflow sensing and gust rejection in biological systems

Flying under wind disturbances outdoors is an ability that animal flyers master, regardless of their size and weight. In doing so they employ multi-modal sensing strategies from their *sensor-rich* system (Mohamed et al., 2014), which is far more sophisticated than the one found in manned or unmanned aerial vehicles.

**FIGURE 1:**
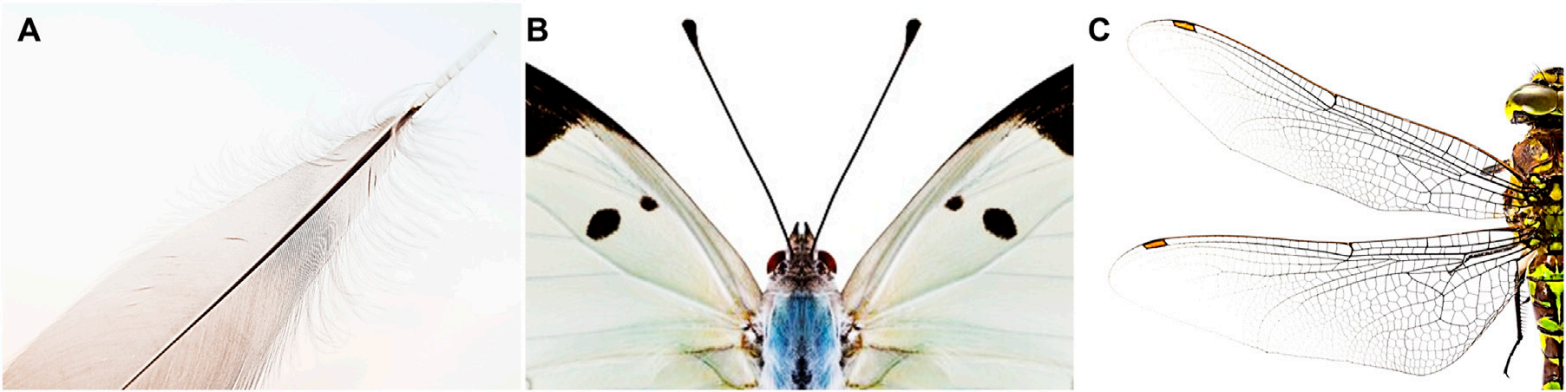
Embodied airflow sensing assets found in Nature. Mechanoreceptors present on, or near, feather follicles **(A)**; antennas or whiskers **(B)**; and distributed on wings **(C)** provide airflow sensing information used by animal flyers to better navigate under wind disturbances. Free-stock images from Pexels (2022).

The family of modern avian species sheds much light on how sensing instrumentation plays an active role in flight control to deal with unknown and disturbed outdoor environments, as illustrated in Figure 1. Among the various corpuscles found in birds, the Herbst corpuscles—nerve endings in feather follicles—are known to serve as airflow and pressure sensors through feather vibration sensing to aid flight control (Hörster, 1990). Brown and Fedde (1993) point out that the mechanoreceptors on or near flying animals’ feather follicles may act like airflow sensors and allow the flying animals to adjust their flapping behavior accordingly.

For insects, their wings contain transducers capable of visual, airflow, inertial, and wing load sensing (Taylor and Krapp, 2007). Fabian et al. (2022) present the first detailed map of the mechanosensor arrays on the dragonfly’s wings through a cross-species survey of sensor distribution with quantitative neuroanatomy. This helps to further understand where and how the sensory apparatus is distributed and integrated into the sensorimotor loop of flight control in insects. More work in this direction on other types of insects, birds or bats would help further unravel the role of airflow sensing in flight control. For example, Sterbing-D’Angelo et al. (2011) provide empirical evidence that hairs on wing membrane support bats with sensory information to respond to changing aerodynamic conditions during flight. The sensory wing hairs located at the wing’s trailing edge are the most sensitive to airflow from the rear, which could aid in reversal airflow detection indicating a higher risk of stall under turbulent conditions. The exact function of the wing hairs in flight control and the associated sensory cells still remain unresolved.

While the direct connection between the sensing packages in avian species and the body control strategy is still ongoing research, studies of birds or insects subjected to different types of wind gusts have proven that these flapping wing animals are aware of the different types of disturbances and use different control strategies accordingly. Jakobi et al. (2018) show that bees execute different control strategies when dealing with gusts from different directions (sideward, upward, or downward directions), overall in the fashion of pitching up and slowing down upon encountering the disturbance. However, the exact role of sensory information on the incoming gust for control mitigation is unclear. Badger et al. (2019) look into how Anna’s hummingbirds fly through upward gusts with the different usages of the wing and tail actuation for enhanced stability and apply such bio-inspiration to improve the stability of a glider undergoing vertical gust. While Gu et al. (2020) investigate fruit flies under headwind gust perturbations and Ravi et al. (2020) study hummingbirds undergoing roll perturbations, they all point out the timing of wing rotations to be critical to mitigating perturbations in both the bird and insect flights. All these works motivate a further investigation into how to improve in-gust flight control given the real-time information of certain types of wind disturbances.

### 1.2 In-gust flight of flapping wing micro aerial vehicles

In-gust flight control and disturbance rejection for micro aerial vehicles (MAVs) has always been a challenge, particularly because aerodynamic forces resulting from gusts have a higher impact on the flight stability of lightweight MAVs than larger aerial vehicles due to lower wing loading. While traditional manned aircraft have employed disturbance rejection schemes for decades, those techniques are often insufficient to be adapted to MAVs due to the highly time-dependent and spatially varying changes in the velocity field (Zarovy et al., 2010).

Several gust disturbance rejection control frameworks for quadrotor platforms have been proposed in the literature; Yang et al. (2017) introduce a dual closed-loop control framework with an extended state observer and active disturbance rejection control in the inner attitude control loop. Tagliabue et al. (2020) use bio-inspired whisker-like airflow sensors to estimate the three-dimensional wind disturbance, the drag force and other interaction force, to further improve the operational safety of the MAV under wind disturbance. In (O’Connell et al., 2022), a deep learning–based trajectory tracking controller enables a quadrotor to learn how to adapt to rapidly changing wind conditions in real time. Furthermore, for fixed-wing MAVs, Castano et al. (2014) first introduce a bio-inspired gust rejection mechanism based on strain sensing feedback to improve roll control during in-gust flights, inspired by the *campaniform sensilla* - strain sensors distributed on insects’ wings (Skordos et al., 2002). Later, Gremillion et al. (2015); Gremillion and Humbert (2015) further expand the disturbance rejection with distributed acceleration and strain sensing to mitigate roll and heave perturbation using the force-sensitive measurements before the disturbance is propagated to lower level states.

As a subset of MAVs, flapping wing MAVs (FWMAVs) have gained renewed interest in recent years for their advanced maneuverability and agility, inspired by their biological counterparts (Ho et al., 2003; Wood, 2008; Keennon et al., 2012; Phan et al., 2017; Karásek et al., 2018; Tu et al., 2020). However, they are more vulnerable to external disturbances such as wind gusts, due to the lower wing loading capabilities with respect to multicopters and fixed-wing platforms.

Several modeling and simulation attempts have been made to evaluate the effects of lateral, vertical, and longitudinal gusts on flapping wing performance. Overall, the flapping wing pair is susceptible to strong downward gusts and has better recoverability in the presence of frontal and side gusts if the gust velocity is less or at the same magnitude as the wingtip velocity (Jones and Yamaleev, 2012). The numerical simulations from (Bhatia et al., 2014) further concur a better tolerance of longitudinal gusts than lateral gusts. Thus, the orientation of the wing stroke plane with respect to the wind gust vector is critical (Jones and Yamaleev, 2016). Flapping wings also show an innate advantage of recovery (Jones and Yamaleev, 2012; Fisher et al., 2016; Nakata et al., 2018) from wind gust fluctuations as the thrust generation returns to the original state within one flapping cycle once the gust no longer affects. A comparative study of the gust mitigation ability of different types of MAVs subject to gusts of the same type is yet to be completed, to further understand and quantify the inherent advantages of utilizing flapping wings.

Extending beyond pure modeling and simulations, only a few experimental attempts to study and develop disturbance rejection methods for the in-gust flight of FWMAVs have been introduced. In (Chirarattananon et al., 2015), disturbance rejection for a continuous frontal wind of 0.6 m/*s* is integrated with both adaptive estimation and least square estimation methods, employed for the RoboBee control (Wood et al., 2013). In (Lee et al., 2020), a disturbance observer-based control (DOBC) is developed on an FWMAV perturbed by lateral wind during flight. Such a learning-based approach is used in conjunction with an *anomaly* detector which enables switching between nominal control and disturbance-rejection control, in the presence of gusts. Both these works validate their approach on a real platform, proving robustness over wind disturbances of a specific nature, i.e. pre-defined constant wind; however both strategies display a reactive approach where disturbances generated by the wind are compensated only after their estimations exceed a certain threshold, in other words with some initial delay. In nature, flying insects adjust their flight attitude and behaviors by directly sensing the wind with their antennae and body hair, and then acting accordingly (Fuller et al., 2014). Furthermore in the work from (Wang et al., 2022), a more active approach is taken towards FWMAVs flight in wind conditions, thanks to the miniaturization of the airflow sensor which could be integrated onboard directly for free flights. The onboard miniature airflow sensor informs a gain-scheduling control approach in the horizontal position control loop to compensate for forward-facing step-increased wind, blowing on the Delfly Nimble platform. The airspeed-enabled gain scheduling approach compensates for positioning errors in the presence of 5 different wind speeds, incremented in steps.

Following upon the above efforts, in this article we introduce a bio-inspired sensing approach to in-gust flight for FWMAVs, combining on-board airflow sensing and an adaptive PID and feed-forward gain scheduling approach for wind disturbance rejection in step-increased wind speeds and alternating-frequency wind speeds. The contributions of this work are: 1) a comprehensive in-gust flight dynamics model for FWMAV that expands upon the effects of frontal wind on the aircraft’s attitude *via* the dihedral angle servo dynamics; 2) aerodynamic drag and thrust models for in-wind flight; 3) an adaptive position and velocity control framework utilizing onboard thermistor-based airflow sensing; 4) validation of the model and control framework in real flight experiments in alternating wind conditions; 5) a performance comparison between our proposed approach and a standard PID controller.

In the following sections of this article, the model of FWMAVs’ in-gust dynamics is firstly derived from an extensive experimental campaign. In this model, the main effects brought by gusts on FWMAVs are captured, namely wind drag forces and dihedral servo control effectiveness reduction. Then, the design of the control framework is discussed and validated with multiple flight experiments under frontal gusts of alternating intensity. Finally, results are analyzed and compared against the non-adaptive control case (standard PID).

## 2 Aerial platform and fan system

**FIGURE 2:**
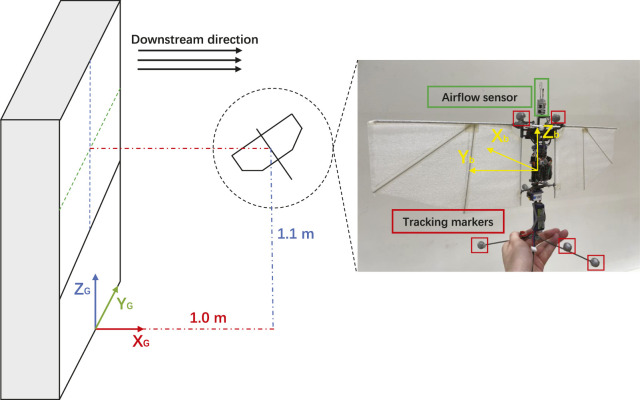
Flapper Drone hovering at a predefined setpoint in front of the fan system, with the onboard airflow sensor mounted on the top of the fuselage. The global coordinate frame originates from the intersection point between the mid-line of the fan system and the ground plane.

The FWMAV platform used is the Flapper Drone^1^, a commercial spin-off from the former research prototype, the Delfly Nimble (Karásek et al., 2018). The platform uses the Crazyflie Bolt flight control suite with an STM32F405 processor. Compared to the DelFly Nimble, the Flapper Drone has a higher mass and wingspan, resulting in a more generous payload and wing loading capacity, as seen in Table 1.To generate the desired dynamic wind conditions in flight experiments, an open-source fan system consisting of an array of 135 axial fans (Olejnik et al., 2022) is employed. With pulse width modulation (PWM) based control of the fans, the fan system can render various types of continuous winds and gusts with an effective wind surface area of approximately 1 m^2^, at flow speeds of different intensities and frequencies. The full experimental setup and used platforms are illustrated in Figure 2.

**TABLE 1:** DelFly Nimble and Flapper Drone specifications.

Parameters	DelFly Nimble	Flapper Drone
Wingspan (cm)	33	49
Takeoff weight (g)	28.2^*^	102^*^, 112.2^*^^*^
Maximum extra payload (g)	∼ 3	∼ 25
Battery capacity (mAh)	180 (LiPo 1S)	300 (LiPo 2S)

^*^min payload, including battery.

^**^including add-on current sensor (Pololu ACS711EX), 9 V step-up voltage regulator (Pololu U3V12F9), the airflow sensor (Modern Device RevP), and associated cables with header pins.

To generate an accurate model of Flapper’s in-gust dynamics flight, an extensive experimental campaign was carried out, and onboard states were measured at different wind parameters. During hovering flight experiments, Flapper was set to hover at a fixed setpoint: 1.0 m downstream from the fan surface of the fan system, 1.1 m above the ground, and the negative direction of body frame axis *X*_*b*_ pointing towards the center of the fan system, ensuring that the platform was fully immersed in the freestream. To log the aircraft position and angular states, the OptiTrack motion capture system was used. Furthermore, additional devices were employed to gather other data, as presented in Table 2.

**TABLE 2:** Overview of data obtained from onboard and offboard sensors during the testing campaign.

Sensor type	Measurements obtained
Optitrack	Position (*x*, *y*, *z*)
	Attitude angles (*ϕ*, *ψ*, *θ*)
Onboard	Linear accelerations (*a*_*x*_, *a*_*y*_, *a*_*z*_)
	Angular velocities (*p*, *q*, *r*)
RevP airflow sensor	Airflow sensing voltage (*V*_*air*_)
Current sensor	Current intensity (*I*)
On-board extra	Dihedral angle command (*γ*_*command*_)
	Dihedral angle output (*γ*_*output*_)
	Battery voltage (*V*_*battery*_)

To generate repeatable gust disturbances of different intensities and frequencies for each flight experiment, the fan system PWM duty cycle has been programmed to stay at rest for the first 10 s, and alternate between a low wind speed value 0.5 m/*s* (PWM duty cycle = 20%) and a high wind speed value 2.1 m/*s* (PWM duty cycle = 60%) or 2.4 m/*s* (PWM duty cycle = 70%) at a fixed frequency during the following 30 s.

## 3 Modelling of flapping wing micro aerial vehicles’s in-gust dynamics

### 3.1 Model structure

Previous studies have modelled the dynamics of FWMAV in the absence of wind, for the purpose of designing new robust control schemes. In (Kajak et al., 2019), a minimal longitudinal model has been proposed for controller design. In (Nijboer et al., 2020), a grey-box longitudinal dynamics model is derived based on free-flight data. Furthermore, in (Bains, 2020), the lateral body dynamics have been modeled with a system identification approach. As these models were derived based on free-flight data with no external wind disturbance, therefore they do not capture the effects of gusts on FWMAV’s system dynamics.

The dynamics of FWMAV’s in-wind flights have also been studied in the past few years. (Chirarattananon et al., 2017) presented an in-wind FWMAV dynamics model consisting of equations of motion and an additional vector *τ*_*w*_ describing the overall wind effects on FWMAV. In (Lee et al., 2020), the attitude dynamics of FWMAV have been modeled by modeling the moments acting on a flying FWMAV.

In both these works, the effects of wind disturbance over the attitude controller have been implicitly considered through the definition of a momentum acting on the aircraft. For our approach, we introduce a more explicit model which considers the in-gust effects on the dihedral servo control effectiveness. The in-wind longitudinal and translational dynamics of FWMAV are defined as follows:

$$m\ddot{x}=Tsin\theta +F_{D_{\mathrm{wind}}}$$ (1)

$$m\ddot{z}=Tcos\theta -mg$$ (2)

Where *m* is the mass, *T* is the thrust generated by the pair of the flapping wings, $F_{D_{\mathrm{wind}}}$ is the wind drag force and *θ* is the body pitch angle.

**FIGURE 3:**
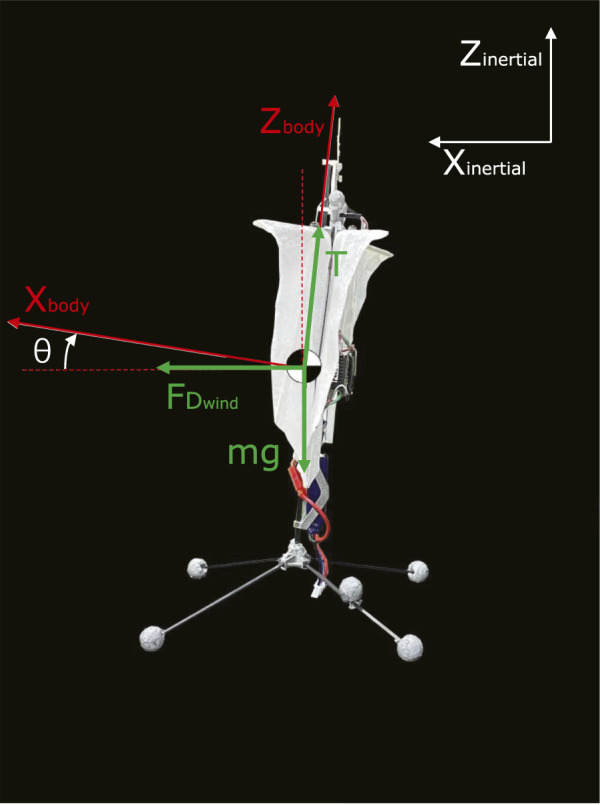
2D longitudinal free body diagram of an FWMAV (side view).

The free body diagram of this model has been shown in Figure 3. Both the thrust force *T* and the wind drag force $F_{D_{\mathrm{wind}}}$ are acting directly on the center of mass (CoM) of the FWMAV. The pitch angle *θ*, which is controlled through the dihedral servo placed near the top of the body’s fuselage, is represented by the angle between the *Z*_*body*_ axis and the *Z*_*inertial*_ axis. To further study and compensate for the wind effects on the FWMAV attitude controller, the in-gust actuator dynamics of the dihedral servo and the wind drag and thrust model are further elaborated in our model in the coming sections.

### 3.2 The effects of wind on dihedral servo control effectiveness

While the Flapper Drone is configured with the X-wing concept similar as the Delfly Nimble, the left- and right-wing pair flaps independently with its own gearbox mechanism capable of flapping frequency modulation through onboard motor speed control. This also allows the generation of variational thrust for the control of roll motion. During forward flight under the impact of the wind from the longitudinal direction, the control of pitch becomes critical as the longitudinal component of the thrust force is in particular susceptible to the wind impact. On the Flapper drone, the pitch motion is generated by changing the dihedral angle (the shifting of the center lines of the flapping wing pairs) through the use of a servo attached to the dihedral arms that connect the fuselage to the left and right wing pairs. Thus, the effective actuation of this dihedral servo becomes interesting to further investigate when the vehicle is under the influence of wind.

**FIGURE 4:**
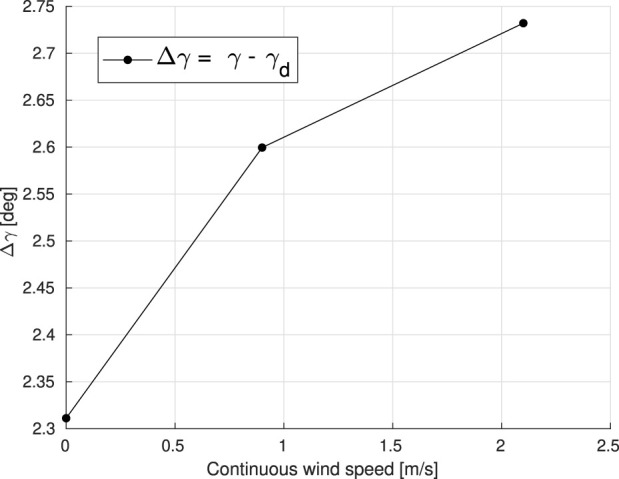
The difference between the actual dihedral angle *γ* and the desired dihedral angle *γ*_*d*_ during stable hover in the frontal wind, at various wind speeds. Each data point in the diagram represents the average over a number of experiments conducted at various wind speeds, namely *V*_*wind*_ = 0, 0.9, and 2.1 m/*s*.

In (Kajak et al., 2019), the effects of forward flight on the dihedral servo of the DelFly Nimble have been shown. Similarly, the Flapper Drone experiences a dihedral servo control effectiveness reduction when hovering under the influence of wind disturbance. In other words, the actual dihedral angle *γ* cannot reach the desired dihedral angle *γ*_*d*_ due to the load imposed by the gusts on the wings. It is noteworthy to mention that the offset in dihedral angle is always present, even at zero wind speed Δ*γ*_(*V*=0)_ ≈ 2.3°. However, such a difference becomes larger at higher wind speeds, as seen in Figure 4.

**FIGURE 5:**
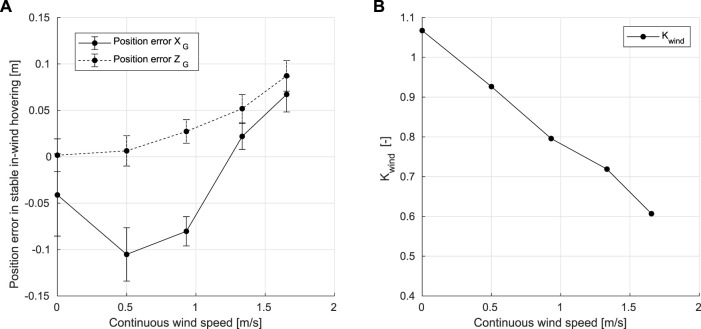
**(A)** Average position errors in *X*_*G*_ and *Z*_*G*_ axis when hovering stably under different continuous wind speeds. **(B)** Values of *K*_*wind*_ under different continuous wind speeds.

To model the dihedral servo control effectiveness reduction, several hovering tests have been conducted with continuous wind speed settings between 0.5 m/*s* and 2.7 m/*s*. The stable hovering positions and pitch angles of the Flapper Drone under different wind intensities are shown in Figure 5. When the wind speed is increasing, the FWMAV moves further downstream with an altitude increase during the stable hovering phase. To compensate for this offset, the dihedral angle *γ* is recomputed as follows:

$$\gamma =K_{\mathrm{wind}}\left(V_{\mathrm{wind}}\right)\;\gamma_{d}\;+\;\mathrm{sign}\left(V_{\mathrm{wind}}\right)C_{\mathrm{corr}}\left(V_{\mathrm{wind}}\right)$$ (3)

where coefficient *K*_*wind*_ is introduced to identify the controller effectiveness reduction, *V*_*wind*_ the wind speed, *γ* the actual dihedral angle output, *γ*_*d*_ the desired dihedral angle, and *C*_*corr*_ the correction term calculated with real-time airflow sensor readings.

To compute *K*_*wind*_, an ordinary least square (OLS) regression method is used based on the collected hovering flight data. *K*_*wind*_ decreases at higher wind speeds, as the control effectiveness reduction becomes more severe, Figure 4. Therefore, referring to the dihedral servo control effectiveness model and stable in-wind hovering positions shown in Figure 5, when the wind speed is increasing, errors in position also increase as the platform moves away from the setpoint, and the control effectiveness reduction will become more severe. To minimize the positioning error, the pitch angle should progressively increase.

### 3.3 Drag model

**FIGURE 6:**
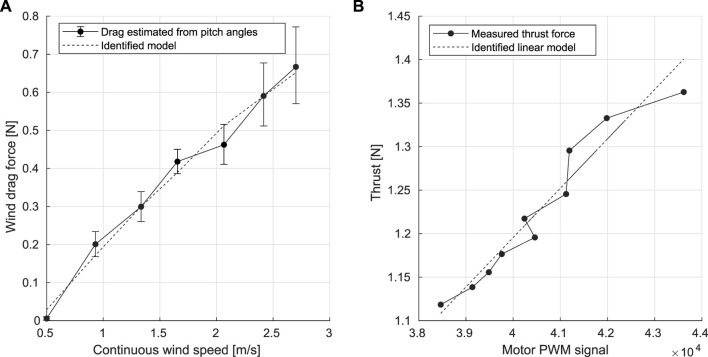
Estimation of wind drag force **(A)** and thrust **(B)**, and the respective identified models.

Based on the composition of forces, the wind drag forces acting on Flapper at different continuous wind speeds have been estimated with the average values of pitch angles and the weight of the Flapper Drone, as seen in Eq. 4.

$$F_{D_{\mathrm{wind}}}=C_{D_{\mathrm{wind}}}V_{\mathrm{wind}}\;\cos\left(\theta \right)$$ (4)

where *C*_*Drag*_ is the drag coefficient and *V*_*wind*_ is the continuous wind speed.Using a polynomial fit over the measured pitch angle data, the drag coefficient has been identified as *C*_*Drag*_ = 0.34. As shown in Figure 6A, the identified model indicates that the wind drag is increasing approximately linearly with the wind speed, which indicates the pitch angle should increase in a similar fashion to compensate for the effects of increased drag and to minimize the position errors.

### 3.4 Thrust model

The thrust model for the Flapper drone was identified through a relationship between the PWM signal sent to the wing motors and the generated lift after attaching known weights on the platform. These experiments allowed to correlate the flapping frequency of the Flapper with the input PWM signals of the wing motors as described by Eq. 5.

$$f_{\mathrm{flap}}=K_{\mathrm{flap}}\;PWM$$ (5)

where *f*_*flap*_ is the flapping frequency, *K*_*flap*_ the conversion coefficient, *PWM* the magnitude of the input PWM signals. Referring to the linear thrust assumptions in (Kajak et al., 2019), the thrust model has been proposed as a linear model in Eq. 6, with both *c*_1_ and *c*_2_ as the fitted coefficients.

$$T=2\left(c_{1}f_{\mathrm{flap}}+c_{2}\right)=2c_{1}K_{\mathrm{flap}}\;PWM+2c_{2}$$ (6)

As shown in Figure 6B, the thrust model has been identified with OLS estimator, in which the thrust force is approximately linear with the magnitudes of the motor’s PWM signal.

## 4 Airflow sensing-based controller design

As previously discussed, the wind directly affects the FWMAV’s in-wind flight through drag and dihedral servo control effectiveness reduction, which renders the FWMAV unable to reach the pre-defined setpoints when attempting to hover stably under the influence of wind disturbance. Based on this situation, a controller is hereby proposed with an adaptive feed-forward (FF) gain in the velocity controller, and an adaptive proportional gain in the position controller to compensate for the dihedral servo control effectiveness reduction, minimize the oscillation along *X*_*G*_ axis and improve the pitch stability when hovering.

**FIGURE 7:**
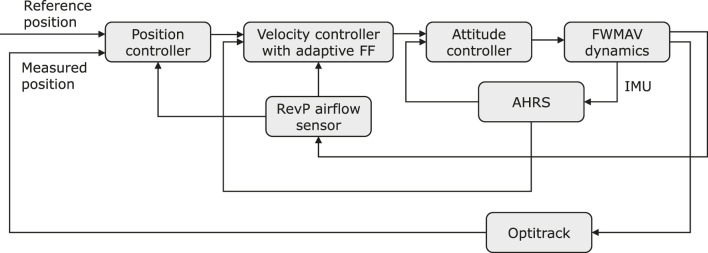
The block diagram showing the implementation of the adaptive-FF controller in FWMAV’s control system.

As shown in Figure 7, the adaptive position controller is implemented based on the error between the actual position (measured *via* Optitrack) and the desired setpoint. The controller also takes into account the airflow sensor readings as the input and computes an updated reference velocity for the velocity controller loop.

Similarly, the adaptive velocity controller is implemented with the measured velocity from the IMU and gyroscope readings, the reference velocity from the position controller, and the airflow sensor reading as its inputs. The output of this controller directly feeds into the attitude control loop, regulating the pitch angle.

### 4.1 Adaptive position controller

**FIGURE 8:**
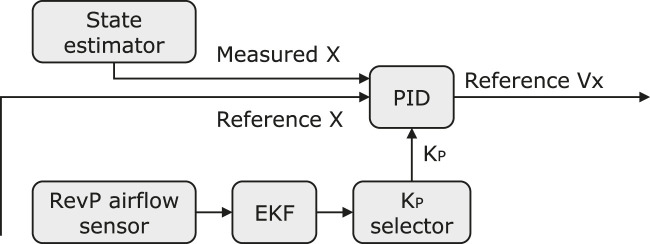
The block diagram of the adaptive position control loop.

Delving deeper into the position controller block of Figure 8, the position error along the *x* axis, *e*_*x*_, is computed, yielding to the reference velocity $V_{x_{\mathrm{ref}}}$ :

$$e_{x}=x_{\mathrm{ref}}-x_{\mathrm{measured}}$$ (7)

$$V_{x_{\mathrm{ref}}}\left(t\right)=K_{P_{x}}\;e_{x}\left(t\right)\;+\;K_{I_{x}}\;\int_{0}^{t}e_{x}\left(\tau \right)\;d\tau \;+\;K_{D_{x}}\;\frac{de_{x}\left(t\right)}{dt}$$ (8)

where $K_{P_{x}}$ is the proportional gain (1.5 in the original position controller), $K_{I_{x}}$ the integral gain (0.0 in the original position controller), and $K_{D_{x}}$ is the derivative gain (0.0 in the original position controller).Rather than the constant $K_{P_{x}}$ value in the original position controller, the value of $K_{P_{x}}$ is changed adaptively by the $K_{P_{x}}$ selector during flights in real time based on the filtered airflow sensor reading. The values of $K_{P_{x}}$ corresponding to different intervals of wind speeds and filtered airflow sensor readings have been shown in Table 3. However, in real flights, the airflow sensor will output unreliable outlier readings occasionally, which results in rapid changes in $K_{P_{x}}$ though the wind speed has not reached certain levels. Therefore, an extended Kalman Filter (EKF) and a $K_{P_{x}}$ selector is employed to filter out the noise and select an appropriate value for $K_{P_{x}}$ . At the time *t* = *t*_*i*_, the $K_{P_{x}}$ selector works as Algorithm 1.

Algorithm 1
$K_{P_{x}}$ and $K_{FF_{x}}$ selector.

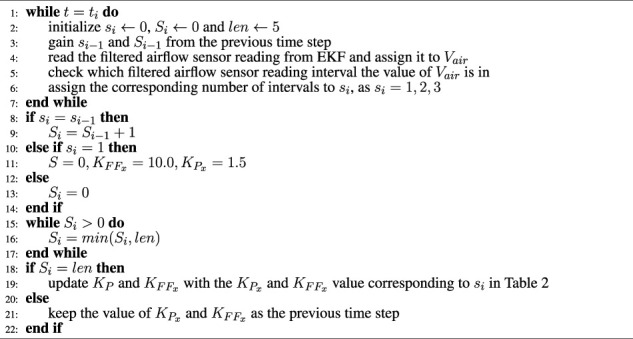

**TABLE 3:** The values of $K_{P_{x}}$ and $K_{FF_{x}}$ with the corresponding ranges of wind speeds and filtered airflow sensor readings.

Wind speed (m/s)	Filtered airflow sensor reading (V)	$K_{P_{x}}$ (-)	$K_{FF_{x}}$ (-)	No. of intervals
(0, 0.780)	(0, 1.740)	1.5 (default)	10.0 (default)	1
(0.780, 1.087)	(1.740, 1.830)	1.65	18.5	2
(1.087, *∞*)	(1.830, *∞*)	1.65	21.5	3

### 4.2 Adaptive feed-forward velocity controller

**FIGURE 9:**
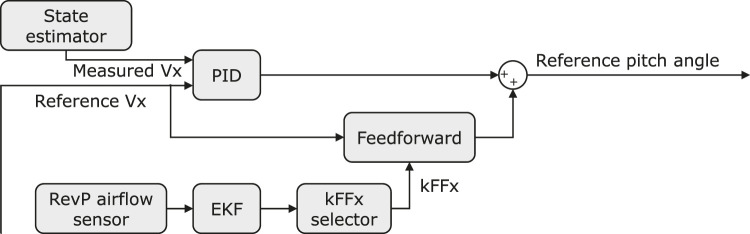
The block diagram of the adaptive velocity controller developed.

As shown in Figure 9, in this adaptive velocity controller, a feed-forward term is calculated based on the reference *X* velocity $V_{x_{\mathrm{ref}}}$ as Eq. 8 and summed with the output from the PID block. Instead of using a constant $K_{FF_{x}}$ as the traditional feedforward controller, the value of $K_{FF_{x}}$ is adjusted actively by the $K_{FF_{x}}$ selector during the flight based on the filtered airflow sensor reading in this adaptive-ff controller.

$$\theta_{FF}=K_{FF_{x}}\;V_{x_{\mathrm{ref}}}$$ (9)

As previously presented, the dihedral servo control effectiveness reduction becomes more severe at higher wind speeds. Therefore, a proportional controller is designed to fuse the information of the onboard airflow sensing to generate higher pitch commands. The values of the proportional gain $K_{FF_{x}}$ correspond to different wind speed regions sensed by the onboard airflow sensor and filtered in real time, as seen in Table 3. Similarly to *K*_*P*_, $K_{FF_{x}}$ also changes rapidly due to the unreliable outlier readings from the airflow sensor though the wind speed has not reached certain levels. Therefore, filtering becomes important to prevent rapidly changing inputs at the controller level. An Extended Kalman Filter (EKF) and a $K_{FF_{x}}$ selector are employed to remove the sensor’s noise and select an appropriate value for $K_{FF_{x}}$ . At the time *t* = *t*_*i*_, the $K_{FF_{x}}$ selector works as explained in Algorithm 1.

Based on the airflow sensor, the adaptive position controller and the adaptive velocity controller, the FWMAV is able to reliably estimate wind speeds in real-time and update *K*_*P*_ and $K_{FF_{x}}$ accordingly in the pitch angle loop and compensate for wind-induced disturbances.

### 4.3 Airflow sensor data filtering

The nonlinear state space model for the Extended Kalman Filter (EKF) is structured as follows with state *x* = *V*_*air*_, the raw readings from the sensor, $\underline{w}\left(t\right)$ the system noise and $\underline{v}\left(t\right)$ the sensor noise.

$$\dot{\underline{x}}\left(t\right)=f\left(\underline{x}\left(t\right),\underline{u}\left(t\right),t\right)+G\left(\underline{x}\left(t\right),t\right)\underline{w}\left(t\right)$$ (10)

$$\underline{z_{n}}\left(t\right)=h\left(\underline{x}\left(t\right),\underline{u}\left(t\right),t\right)$$ (11)

$$\underline{z}\left(t_{k}\right)=\underline{z_{n}}\left(t_{k}\right)+\underline{v}\left(t_{k}\right)$$ (12)

To improve the computation efficiency and reduce latency for real flights, the EKF algorithm has been simplified as follows. Firstly, since the time derivative of the state $\dot{x}\left(t\right)={\dot{V}}_{\mathrm{air}}$ is only related to the stochastic system noise $\underline{w}\left(t\right)$ , the state function $f\left(\underline{x}\left(t\right),\underline{u}\left(t\right),t\right)$ is taken as 0 and the system noise input function $G\left(\underline{x}\left(t\right),t\right)$ is set as 1. Secondly, in this EKF only the sensor reading *V*_*air*_ is to be observed, which results in the observation equation $\underline{z_{n}}\left(t\right)$ being simplified as $\underline{z_{n}}\left(t\right)=\underline{x}\left(t\right)={\underline{V}}_{\mathrm{air}}$ . The state and the observation model have been simplified as:

$$\dot{\underline{x}}\left(t\right)=\underline{w}\left(t\right)$$ (13)

$$\underline{z_{n}}\left(t\right)={\underline{V}}_{\mathrm{air}}$$ (14)

$$\underline{z}\left(t_{k}\right)=\underline{z_{n}}\left(t_{k}\right)+\underline{v}\left(t_{k}\right)$$ (15)

The system noise covariance *Q* and the sensor noise covariance *R* are estimated as *Q* = 0.001 and *R* = 0.01. Together with the initial state of $\underline{x}\left(0\right)$ set as 1.433 and the initial guess of covariance of state estimation error *P*_0,0_ as 0.0001, the EKF is implemented to filter out the noise in the airflow sensor readings.

## 5 Free-flight experiments

### 5.1 In-gust hover flights with PID control

**FIGURE 10:**
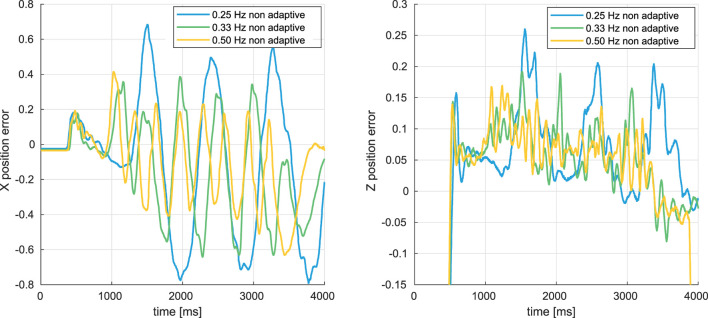
Time histories of X and Z position errors during the alternating-wind experiments, with the original PID controller.

Several in-gust hovering flights have been conducted with the original PID controller under alternating wind speeds between 0.5 m/*s* and 2.4 m/*s*, and between 0.5 m/*s* and 2.1 m/*s*, at the frequency of 0.25 *Hz*, 0.33 *Hz*, 0.50 *Hz* and 0.75 *Hz*. The time histories of the position errors in *X*_*G*_ and *Z*_*G*_ axes during alternating wind speeds between 0.5 m/*s* and 2.4 m/*s*, are shown in Figure 10.

As shown in Figure 10, the FWMAV oscillated greatly under these dynamic gusts. From Figure 12, the RMS errors of both *X*_*error*_ and *Z*_*error*_ are increasing, which indicates that the position control is degrading and the oscillation becomes more and more severe when the changing frequency of the gust *f*_*gust*_ is decreasing since within one period the gust of high wind speed always lasts longer (from 0.75 s to 2.00 s) before decreasing to 0.5 m/*s*.

Furthermore, when the maximum wind speed increases from 2.1 m/*s* to 2.4 m/*s*, the oscillation also becomes more severe because the gust intensity is increasing.

### 5.2 In-gust hover flights with adaptive PID control

**FIGURE 11:**
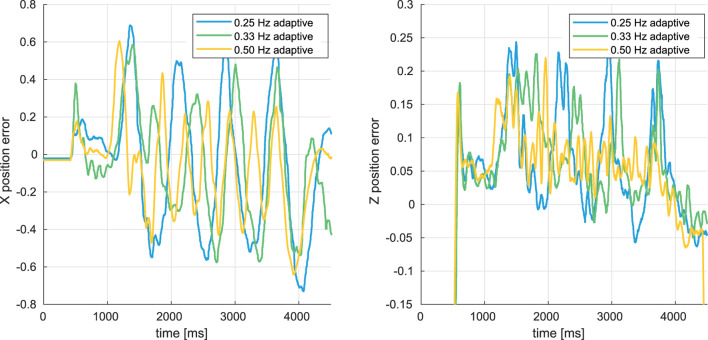
Time histories of X and Z axis position errors during the in-gust hovering flights with adaptive position and velocity controller.

To validate the adaptive position and velocity controller, several in-gust hovering flights have been conducted under the gusts alternating the wind speed between 0.5 m/*s* and 2.4 m/*s*, and between 0.5 m/*s* and 2.1 m/*s* at the frequency of 0.25 *Hz*, 0.33 *Hz*, 0.50 *Hz* and 0.75 *Hz*. The time histories of position errors in *X*_*G*_ and *Z*_*G*_ axis from the flights under the gusts changing the wind speed between 0.5 m/*s* and 2.4 m/*s* have been shown in Figure 11.

**FIGURE 12:**
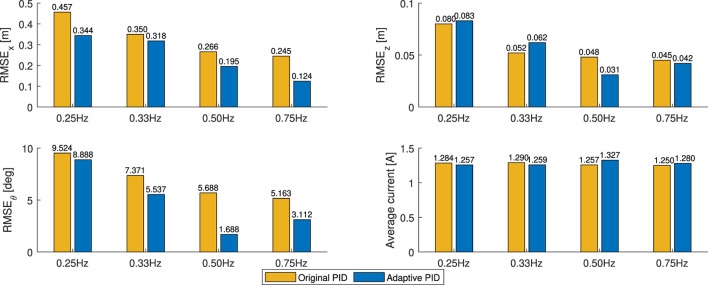
Average values of position errors in *X*_*G*_ axis and current intensities during the in-gust hovering flights under the gust changing between 0.5 m/*s* and 2.4 m/*s*.

**FIGURE 13:**
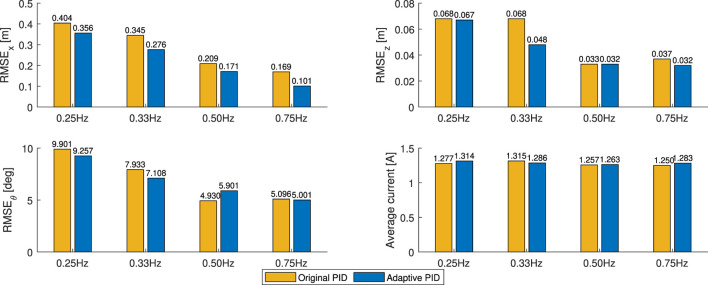
Average values of position errors in the *X*_*G*_ and *Z*_*G*_ axes, and current drawn during the in-gust hovering flights under alternating winds between 0.5 m/*s* and 2.1 m/*s*.

Similar to the in-gust flight experiments in Section 4.3, the FWMAV is oscillating during these in-gust hover flights. As presented in Figures 12, 13, when the gust changing frequency *f*_*gust*_ is decreasing and the maximum wind speed increases from 2.1 m/*s* to 2.4 m/*s*, the oscillation is becoming more and more severe.

## 6 Performance analysis and comparison

The root mean square error (RMSE) of the *X*_*G*_ position, the *Z*_*G*_ position and pitch attitude angle *θ* from in-gust flights with both the original PID controller and the adaptive PID controller are shown in Figures 12, 13, together with the average drawn current during flight.

As shown, the RMSE value of the *X*_*G*_ position *RMSE*_*X*_ decreases when the alternating gust frequency decreases. Comparing with the original PID controller, *RMSE*_*X*_ values of the cases with the adaptive PID controller are always lower, indicating better performance in disturbance rejection and a robust response along the *X*_*G*_ direction in the position controller. Furthermore, the RMSE values of the pitch attitude angle *RMSE*_*θ*_ of the adaptive PID cases are also always lower than the cases with the original PID controller, except for the case of alternating winds between 0.5 m/*s* and 2.1 m/*s* at 0.33 *Hz*, indicating that the pitch stability for FWMAV’s in-gust flight has been improved.

Though this adaptive PID controller focuses mainly on reducing the position error along the *X*_*G*_ direction, along the *Z*_*G*_ direction, the RMSE values of the position error *RMSE*_*Z*_ are lower than the cases of original PID controller in the high gust changing frequency cases (0.75 *Hz*) and remain the similar magnitudes in the lower cases (0.25 *Hz* and 0.33 *Hz*). Furthermore, for the flights with the adaptive PID controller, the average in-flight current intensity values are slightly higher than the cases with the original PID controller when the gust changing frequencies are higher (0.50 *Hz* and 0.75 *Hz*) and are slightly lower than the cases with the original PID controller when the gust changing frequencies are lower (0.33 *Hz*), which indicates the energy consumption levels remain similar in these in-gust flights.

**FIGURE 14:**
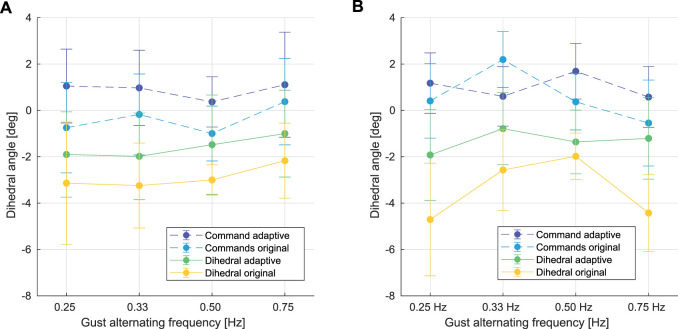
Comparison between dihedral commands from the flights with the adaptive PID controller (dark blue), dihedral commands from the flights with the original PID controller (light blue), dihedral outputs from the flights with the adaptive PID controller (green), and dihedral outputs from the flights with the original PID controller (yellow) during the in-gust hovering flights under the gust alternating between 0.5 m/*s* and 2.4 m/*s* **(A)**, and between 0.5 m/*s* and 2.1 m/*s* **(B)** at different gust frequencies.

Furthermore, the increasing offset in dihedral angle error during the in-gust flights is reduced when the proposed adaptive controller is employed. This demonstrated the effectiveness of our adaptive control approach, which renders a better performance than the original PID controller, as shown in Figure 14.

## 7 Conclusion

In this article, two main aspects contributing to the wind-induced disturbances on FWMAVs are identified, namely the wind drag force and the dihedral servo control effectiveness reduction. An expanded in-gust dynamics model is presented, which includes the dihedral servo dynamics and accurate thrust and drag models for the platform of study, the *Flapper* drone. A bespoke, adaptive PID controller is implemented for both the position and velocity control loops, fusing the onboard airflow sensing information after filtering. The novel control framework is then validated in several flight experiments under alternating gust conditions, at four different frequencies. The proposed controller’s performance is analyzed against the standard cascaded PID controller, proving that our method with active airflow sensing is able to damp the oscillations in the *X*_*G*_ direction by 25.15% and improve the pitch stability efficiently when the drone is subjected to frontal gusts of alternating wind speeds up to 2.4 m/*s*. At the same time, we demonstrate the energy efficiency of our controller by proving that equal current is drawn in flights, despite the higher commanded pitch values in the attitude control loop.

Future work will investigate the possibility to design a similar adaptive controller for the thrust loop of the FMWAV, which could improve the position control along *Z*_*G*_ axis and further improve the energy efficiency. A better estimator for current wind speed could also be designed and implemented with the airflow sensor to replace the Extended Kalman Filter used in the adaptive PID controller. Furthermore, to remove the steady-state error, an integral gain could be introduced in the position controller in Figure 7.

## Data Availability

The datasets presented in this study can be found in online repositories. The names of the repository/repositories and accession number(s) can be found below: https://github.com/asukaRyouji/Adaptive-Flapper-Datasets.

## Footnotes

1
https://flapper-drones.com/wp/
